# The Implication of Sphingolipids in Viral Infections

**DOI:** 10.3390/ijms242417303

**Published:** 2023-12-09

**Authors:** Sanya Thomas, Stephen Varghese Samuel, Annmarie Hoch, Caitlin Syphurs, Joann Diray-Arce

**Affiliations:** 1 Precision Vaccines Program, Department of Pediatrics, Boston Children’s Hospital, Boston, MA 02115, USA; sanya.thomas@childrens.harvard.edu (S.T.); annmarie.hoch@childrens.harvard.edu (A.H.); caitlin.syphurs@childrens.harvard.edu (C.S.); 2 Harvard Medical School, Boston, MA 02115, USA; stephenvarghesesamuel@gmail.com; 3 Department of Emergency Medicine, Christian Medical College and Hospital, Vellore 632004, India

**Keywords:** sphingolipids, sphingosine-1-phosphate, lipid metabolism, sphingolipid metabolism, viral infections

## Abstract

Sphingolipids are involved in cell signaling and metabolic pathways, and their metabolites play a critical role in host defense against intracellular pathogens. Here, we review the known mechanisms of sphingolipids in viral infections and discuss the potential implication of the study of sphingolipid metabolism in vaccine and therapeutic development.

## 1. Introduction

Sphingolipids are crucial components of eukaryotic cell membranes. Common types of sphingolipids include sphingosine, ceramide, sphingosine-1-phosphate (S1P), and ceramide-1-phosphate (C1P) [1]. Sphingolipids are amphipathic molecules comprising of hydrophobic and hydrophilic regions. The hydrophobic end consists of a sphingoid long chain base to which fatty acid is attached at the second carbon atom by an amide bond, forming ceramide [2]. Typically, the sphingoid long chain base includes sphingosine, sphinganine, or phytosphingosine. Sphingolipids vary based on the type of sphingoid bases, fatty acids, length of fatty acid chain, degree of saturation, degree of hydroxylation, sphingoid base, and sugar moiety added to ceramide [1].

The action of sphingolipids on protein targets like kinases, phosphatases, lipases, and membrane receptors renders them with distinct cellular functions involving growth regulation, cell migration, adhesion, apoptosis, senescence, and inflammatory response [1]. They are involved in immunity, neurodegenerative processes, metabolic disorders, cancers, cardiovascular disorders, and skin integrity. Impaired sphingolipid metabolism is linked to major human diseases as these molecules play a critical role in cell signaling pathways [1]. The term ‘sphingolipidosis’ was coined to describe the pathological changes in sphingolipid metabolism resulting in human diseases commonly known as lysosomal storage disorders. Sphingolipids serve as receptors for viral entry and modulators for viral replication [3]. Development of strategies to manipulate the sphingolipid metabolism in the host may inhibit viral replication, contributing to antiviral immune response [4].

In this review, we aim to provide an overview of the biosynthesis and mechanism of action of sphingolipids and the role they play in viral infections, and propose future directions for research that may bridge current gaps in the prevention and treatment of infectious diseases. 

## 2. Biosynthesis of Sphingolipids

**Figure 1:**
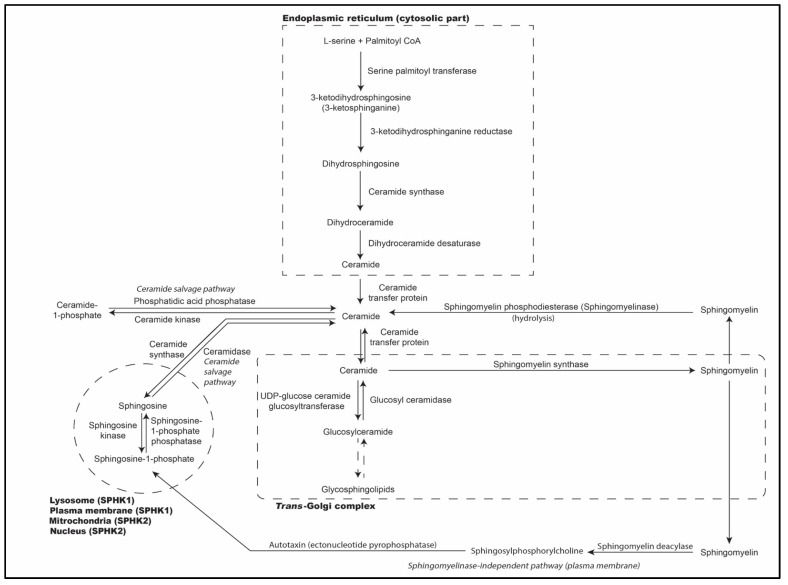
Sphingolipid biosynthesis.

As illustrated briefly in Figure 1, sphingolipids are synthesized in the endoplasmic reticulum through the condensation of an amino acid, commonly serine amino acid, with a fatty acid by reaction with coenzyme-A (CoA) [5]. This is catalyzed by serine palmitoyl transferase (SPT) and is the first and rate-limiting step in sphingolipid synthesis, which results in the generation of 3-keto-dihydrosphingosine (3-KDS) [6,7,8]. 3-KDS is reduced by 3-KDS reductase to form dihydrosphingosine [9]. The fatty acid tail, palmitoyl CoA, is added to the sphingoid base, sphingosine, to form dihydroceramide [10,11]. A cis 4–5 double bond is attached to this sphingoid base to form ceramide, the precursor of different sphingolipid metabolites such as sphingosine, S1P, C1P, sphingomyelin, and glycosphingolipids [1,12]. It is converted to cerebrosides by glucosylceramide synthase by adding UDP-glucose or UDP-galactose, the active forms of glucose and galactose. When UDP-sugars and CMP-sialic acid, like N-acetyl neuraminic acid, are attached to the cerebroside, ganglioside is formed. Ceramide is also converted to sphingomyelin by the enzyme sphingomyelin synthase [1,13]. Phosphatidylcholine acts as the choline phosphate donor in sphingomyelin synthesis [1,8,14,15]. Regulation of sphingolipid metabolism occurs at multiple levels and includes control of enzyme expression, post-translational modification, and allosteric mechanisms [14,15].

SPT, the key regulatory enzyme involved in sphingolipid synthesis and metabolism, is encoded predominantly by the *SPTLC1* gene on chromosome 9q22.31 and *SPTLC2* or *SPTLC3* genes [1]. The catalytic subunits of SPT (SPT1 and SPT2) are responsible for regulating metabolic flux and condensation of L-serine and palmitoyl CoA [2,16,17]. The regulatory subunits of the SPT complex in humans include ORMDL1, ORMDL2, and ORMDL3 [18]. The binding of ceramide with the SPT-ORMDL complexes, resulting in their inhibition, is critical for sphingolipid homeostasis in the cells [19]. Since human ORMDLs lack the canonical phosphorylation site, regulation of SPT may not be directly dependent on phosphorylation [18,19].

## 3. Role of Sphingolipid Metabolites in Immunometabolism

Ceramides, a class of sphingolipids, act as an intracellular second messenger for TNF_α_, IL-1_β_, and other cytokines [20]. Their levels are sensed by the protein family ORMDL that inhibits SPT through dephosphorylation of ORM, causing downstreaming of the target of rapamycin complex-2 (TORC-2) [21,22,23]. Cells sense S1P through the S1PR/ORMDL axis for maintaining sphingolipid homeostasis [24]. Ceramide synthases (CerS) are activated by deacetylation via NAD-dependent protein deacetylase sirtuin-3 and mitochondrial SIRT-3 and by phosphorylation via casein kinase 2 [25]. Inhibition of CerS contributes to ceramide’s role in cellular stress response, including heat shock [20]. Inhibition of dimerization of CerS-2 and CerS-6 by BCL-2-like protein 13 results in the cessation of apoptosis [26].

Sphingolipid metabolites, such as sphingosine and S1P, are involved in calcium homeostasis, cellular proliferation, and apoptosis [20]. S1P, generated via the sphingosine biosynthesis pathway by sphingosine kinase-1 or 2 (SphK1 or SphK2), additionally regulates lymphocyte departure into circulation via S1P receptor 1 (S1PR1) signaling [27]. S1PR1 belongs to a family of five G-protein-coupled receptors that mediate cell growth, proliferation, and/or survival [27]. S1P is known to control the differentiation of regulatory T cells (Tregs) and T helper-17 (Th17) cells during immune cell activation, alongside modulating T cell trafficking [27,28]. The reciprocal differentiation of Th1 and Treg cells is theorized to be directed by the S1PR1-mTOR axis, as S1PR1-induced selective activation of the protein kinase B (Akt)/mammalian target of rapamycin (Akt-mTOR) kinase pathway impedes the development and function of Tregs [29].

SphK1 is activated by phosphorylation and causes growth factor-mediated production of phosphatidic acid by downregulating molecules such as protein kinase C and phospholipase D. The property of SphK1 to sense anionic positive membrane curvatures aids endocytosis [30]. Previous studies have demonstrated that sphingolipid metabolism induces immune response and inflammation by creating an S1P gradient in which lymphocytes exit the low-S1P environment in lymphoid organs and tissues where T cells are initially activated and enter the high-S1P environment of bloodstream and circulatory fluids, primarily using S1PR1 [31,32]. Further immunometabolic research suggests a direct correlation and role of SphK1-generated S1P in peripheral T cells through the regulation of peroxisome proliferator-activated receptor gamma (PPAR_γ_), a lipid transcription factor that suppresses lipolysis-dependent energy generation and regulates lipolysis in T cells [27]. During an immune response, activated T cells express increased levels of SphK1, resulting in increased concentrations of intrinsic S1P—which is consistent with an observed increase in S1P concentrations and reduction in surface S1PR1 among lymph node cells following an immune response [32,33].

## 4. Role of Sphingolipids in Infectious Diseases

Sphingolipids are important signaling mediators that play a critical role in cell survival and stress response in addition to regulating inflammatory responses [34]. Perturbations in sphingolipid metabolism play a key role in inflammation in response to bacterial and viral infections [31]. Altered sphingolipid metabolism in the alveolar compartment of the lungs leads to inflammatory responses and elevated ceramide levels leading to pathological hyperinflammation [34]. This heightened sensitivity of the human respiratory system to sphingolipid alteration underscores its pivotal role. 

The action of S1P is required for the egress of lymphocytes out of lymphoid organs. S1PR1 emerges as a major regulator of immunity in both adaptive and innate immune responses to infectious diseases. It is expressed ubiquitously across cells for supporting key roles in cellular functions during an innate immune response, recruiting and trafficking of innate immune cells, macrophage polarization, and plasmacytoid dendritic cell function [28].

## 5. Knowledge Gaps in Sphingolipid Metabolism

Sphingolipids play a key role in intercellular interaction and recognition; however, the clinical applications of their mechanisms remain a scientific mystery to date. Understanding the interaction between S1P receptor signaling and the innate immune response is valuable for understanding its implication in precision medicine as little is known about how changing S1P levels may affect immune responses [32]. Inflammatory monocytes (iMo) play an important role in S1P concentrations by regulating both S1P receptors and S1P gradients to ensure the presence of immune cells at the inflammation site [32]. iMo relies on CD69 to supply S1P in lymph nodes as CD69 is upregulated by T cells and is expressed robustly after an immune response [32]. However, the function of CD69 in the immunometabolic pathway of S1P remains unclear [32]. Research on the essential role of S1P metabolism in immunity is warranted. 

## 6. Sphingolipids as Potential Targets against Viruses

**Figure 2:**
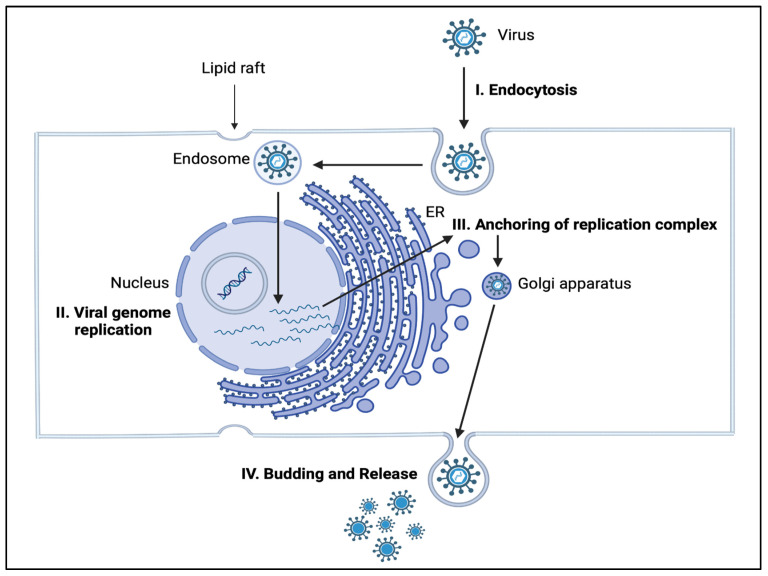
Potential targets in sphingolipid metabolism for viral infections. Potential targets include the cell surface (alteration in lipid composition aiding viral entry), nucleus (alteration of replication machinery), lysosome (inhibition of degradation), endoplasmic reticulum (anchoring of replication complex), and the cell membrane (viral budding and release). The lipid raft in the cell membrane is composed of sphingolipids and glycerophospholipids, and it promotes receptor-mediated endocytosis when a virus binds to it. The nucleus contains sphingolipids that aid in the replication of the viral genome. ER: endoplasmic reticulum.

The survival of enveloped RNA viruses, such as influenza, human immunodeficiency virus (HIV), hepatitis C virus, and dengue virus, depends on host lipid biosynthesis. The lipid bilayer envelope of the RNA virus acts like a protective covering (capsid) that preserves the genetic code in the RNA and is essential for its survival and replication. The RNA becomes vulnerable if the integrity of the capsid is lost. The lipid composition of the capsid includes phospholipids, sphingolipids, and cholesterol which may vary between RNA viruses. When a virus encounters the host surface, it attempts to dock to the surface via interactions between viral envelope glycoproteins and cellular receptors where the proportion of lipids is high. Once the virus enters the host cell, it takes control of host metabolism, particularly lipid metabolism, to make new viral particles (Figure 2) [35,36,37]. Table 1 and Table 2 provide a summary of the mechanisms of action of select viruses and their effects on target sphingolipids.

Although enveloped RNA viruses can attach to the host’s lipid membrane, they require distinct host membrane characteristics to enter the host cell due to the polarity of their genome. Positive-stranded RNA viruses replicate in the cytoplasm, whereas negative-stranded RNA viruses like influenza virus and HIV replicate in the nucleus of the host cell. This enables them to evade innate host immune responses [38,39,40,41,42,43,44].

### 6.1. Influenza Virus

Influenza virus is one of the leading causes of hospitalization and death worldwide. It is a single-stranded enveloped virus with three major sub-viral components: an outer layer consisting of a viral envelope with three transmembrane proteins (hemagglutinin, neuraminidase, M2 protein), an intermediate layer of matrix protein (M1), and an innermost viral ribonucleocapsid core consisting of nucleoprotein (NP) and RNA viral strand (vRNA) [45]. Hemagglutinin and neuraminidase are envelope spike glycoproteins that aid in the docking of the virus to the host cell surface, mediating entry and release of the virus in addition to functioning as the antigenic determinant.

Sphingomyelin synthase-1, present in the Golgi apparatus on the luminal side of the trans-Golgi network and in distal Golgi regions, catalyzes the transfer of the phosphocholine group from phosphatidylcholine onto ceramide to form sphingomyelin [46,47]. In host cells where sphingomyelin synthase-1 is deficient or blocked by SPT inhibitor (myriocin), there is reduced host cell expression of surface glycoproteins, resulting in inhibition of viral docking on host cell—thus reducing the infectivity of the virus [48,49,50]. Deficiency of sphingomyelin synthase-1 causes impaired membrane organization and trafficking of hemagglutinin [51]. The accessibility of hemagglutinin and neuraminidase to the enzyme sortase is blocked in sphingomyelin synthase-1-deficient cells and Madin-Darby canine kidney cells exposed to myriocin, resulting in the inhibition of viral particle release from infected cells [48]. The influenza virus buds from the host cell plasma membrane, from the basolateral and apical domains of a polarized epithelial cell. The apical membrane was found to be studded with sphingolipids on quantitative shotgun mass spectrometry of influenza virus-infected Madin-Darby canine kidney cells [52].

The characteristics of the membrane from which newly formed viral particles originate are determined by certain classes of sphingolipids that target signals carried by viral membrane glycoproteins [48]. Lipid rafts are focal lipid concentrations of cholesterol and sphingolipids (sphingomyelin and glycosphingolipid) on the membrane, which give the cell membrane resistance to extraction by non-ionic detergents and alter membrane behavior [53,54]. Viral infectivity was found to be reduced when hemagglutinin fails to associate with lipid microdomain [49]. The influenza virus depends on the sphingomyelin biosynthesis pathway for entry into host cells [48]. The sphingolipid glucosylceramide can increase the uptake of influenza virus by the cell [55]. The virus uses glucosylceramide for endocytic trafficking and can be decreased by restricting the amount of its metabolizing enzyme, glucosylceramidase, present in the cell. This study found similar results with the Ebola virus, vesicular stomatitis virus, and measles virus [55].

Sphingosine kinase-1, which catalyzes the step that converts sphingosine into S1P, was found abundantly in cells infected with influenza virus; it plays a critical role in exporting viral proteins from the nucleus to the cytoplasm. Blockade of sphingosine kinase-1 hampers viral RNA replication, reducing infectivity [50]. The presence of sphingomyelinase in the cell can suppress viral replication and prevent infection as sphingomyelins are downregulated in the presence of the virus [56].

After replication, the virus uses the lipid-raft microdomains densely packed with sphingomyelin and cholesterol on the host cell surface to assemble the newly synthesized viral particles and initiate the budding process. Perturbation of sphingomyelin synthesis on the host cell membrane can adversely affect the trafficking of viral hemagglutinin and neuraminidase to the host cell surface. This interferes with viral growth, budding, and release—thus reducing infectivity [48].

### 6.2. Dengue Virus

Dengue virus causes a prevalent arboviral disease that is transmitted by mosquitoes to humans. It makes use of sphingolipid metabolism for its replication [4]. It upregulates the expression of ceramide and sphingomyelin in mosquito cells and causes lipid accumulation in the cell membrane fraction enriched in the viral replication complex that may affect virus-induced intracellular membrane architecture [57]. A few animal studies were conducted to address the influence of glycosphingolipid on dengue virus infection [37,53].

Certain plasma trans-membrane protein receptors in phagocytes—T-cell immunoglobulin domain and mucin domain (TIM) and Tyro-3, Axl, and Mer (TAM), which are dengue viral entry factors—were found to interact with phosphatidylserine-dependent phagocytic engulfment and removal of apoptotic cells [37]. A mouse melanoma cell (B16 cells) and its cell counterpart deficient in glycosphingolipid (GM95 cells) showed resistance to dengue infection in GM95 cells when compared to B16 cells, showing that dengue virus requires glycosphingolipid GM3 for viral genome replication and pathogenesis [53]. Glycoprotein GM3 is present in the plasma membrane of host cells, and its concentrations were found to be elevated in dengue-infected cells. Soyasaponin-1-induced inhibition of the synthesis of glycoprotein GM3 showed reduced dengue viral replication in mice brains at days 2 and 4, and it improved survival rates in dengue-infected suckling mice [53].

Vaccines and drugs targeting glycoprotein GM3 could aid in treating infected patients, in addition to reducing the overall burden of dengue virus in endemic areas [13].

### 6.3. Zika Virus

Zika virus, an enveloped positive-strand RNA neurotropic virus, utilizes host machinery for replication and infectivity [54]. Elevated levels of ganglioside antibodies (antibodies against GM2, GM1, GA1, and GD1) were found in 42 patients with acute inflammatory demyelinating polyneuropathy and acute motor axonal neuropathy variant infected with Zika virus [58]. In a recently published global lipidomic survey, a lipid network map was created during an outbreak of Zika virus which found that the NS4B protein of the virus altered host sphingolipid composition aiding viral entry and elevated ceramide levels were redistributed to replication sites, thereby sensitizing cells to Zika virus infection. Blocking the sphingolipid pathway protects against Zika virus via ceramide flux—a key mediator of Zika infection [59]. A recent study demonstrated that Zika virus requires host glycosphingolipids for viral replication [60]. The knockout of glucosylceramide and lactosylceramide synthase encoding genes in this study showed a reduction in viral titers. Additionally, the inhibition of glucosylceramide synthase significantly reduced viral genome replication [60].

### 6.4. Japanese Encephalitis Virus (JEV)

Japanese encephalitis, a mosquito-borne severe debilitating neurological disease endemic in the Asian subcontinent, is caused by JEV. The RNA core of the virus is covered by a lipid bilayer envelope [58]. Replication of JEV depends on sphingomyelinase activation. Sphingomyelinase depletes surface sphingomyelin to ceramide, causing increased infectivity by propagation of JEV [61]. Sphingomyelin synthase 1 deficiency was also found to reduce JEV load in the cells after infection, suggesting an association between sphingomyelin synthase 1 present on the cell membrane and JEV attachment and infection [62]. Sphingomyelinase inhibitors or molecules against sphingomyelin synthase 1 can be a potential therapeutic target in combating Japanese encephalitis in endemic regions [61].

### 6.5. West Nile Virus

West Nile virus causes a neurotropic arthropod-borne disease. Sphingomyelin level in a cell increases West Nile virus infectivity by increasing West Nile virus replication platforms of ds-RNA at cytoplasmic foci. West Nile virus replicates at a rapid rate in mice deficient in acid sphingomyelinase due to its inability to catabolize sphingomyelin. In patients with Niemann-Pick disease type A, where sphingomyelin accumulates in cells due to the inability to catabolize sphingomyelin, enhanced replication of West Nile virus was seen [63]. DS609 and SPK601 are pharmacological therapies targeted at inhibiting sphingomyelin synthesis, thereby reducing infectivity by reducing the release of West Nile virus from infected cells. However, they did not reduce the release of the viral genome. Further studies also showed that inhibition of neutral sphingomyelinase activity hampered the release of West Nile virus particles [64].

### 6.6. Hepatitis C Virus

Hepatitis C is an enveloped positive-stranded RNA virus that is responsible for hepatitis and chronic liver disease. Its replication occurs in a lipid raft membrane [65,66]. The hepatitis C virus is enriched with lipids, particularly sphingolipids, and they play a crucial role in viral entry and genome replication [13,67]. Abnormalities in the glycosphingolipid metabolism (salvage pathway) and the sphingomyelin-sphingosylphosphorylcholine-S1P pathway contribute to disease pathogenesis [5]. In a study where the hepatitis C virus depleted sphingomyelin by hydrolysis, internalization of the virus into host cells was hindered, preventing Hepatitis C infectivity [68]. In another study, Hepatitis C virus infection led to an increase in sphingomyelin production, resulting in increased sphingomyelin-induced viral replication via RNA-dependent RNA polymerase. This produces a “membranous web” in the cytosolic membrane vesicles which utilizes viral and host factors to amplify and sustain viral genome replication via redirection of four-phosphate adaptor protein-2 (FAPP-2), a glycosphingolipid protein carrier with two lipid-binding domains for phosphatidylinositol 4-phosphate and glycosphingolipids, to the “membranous web” where viral replication is active [69,70,71]. Alteration of lipid raft composition, antibodies against sphingolipid biosynthesis, and molecules against FAPP-2 lipid binding sites could serve as potential therapeutic targets [68].

### 6.7. Ebola Virus

Ebola virus has been shown to use sphingomyelins for cell binding and promoting infection, with depletion of sphingomyelins in the cell reducing infection [72]. Acid sphingomyelinase, used to generate sphingomyelins, was shown to play a critical role in Ebola infection; its migration to the cell surface being a key component for infection via exocytosis. More work is needed to generate definitive conclusions, but this indicates that acid sphingomyelinase, and subsequently sphingomyelins, could play a vital role in viral entry. 

### 6.8. Norovirus

Norovirus, a positive-stranded RNA virus, requires an SPT complex to synthesize sphingolipids for internalization into host cells. Sptlc 1 and Sptlc 2, members of the SPT complex, are required for the replication of murine norovirus [73]. Ceramide-enriched membrane domains and CD300LF protein, present on the cell surface, undergo conformational changes for viral binding [74]. Further research into the mechanism of action of the SPT complex in human norovirus infection could identify potential targets for vaccine development.

### 6.9. Adenovirus

Adenovirus interacts with receptors on the host cell surface, CAR, and avB3 integrins, leading to the exposure of protein IV on the viral surface. This results in calcium ion influx via the membrane and subsequently exocytosis by the lysosomes, a membrane repair process that releases acid sphingomyelinase. Sphingomyelin is degraded to ceramide derivatives in the plasma membrane, which are enriched on cell membrane microdomains, promoting viral endocytosis and accumulation of lytic protein VI. This aids in endosomal breakage and the release of viral capsid into the cytoplasm. A positive feedback loop is observed between adenoviral uncoating and lipid signaling for membrane penetration [75]. Genomic interference at the RNA level or molecular inhibition of acid sphingomyelin degradation can hinder endocytosis, resulting in reduced infectivity [75].

### 6.10. Human Immunodeficiency Virus (HIV)

HIV causes preventable acquired immunodeficiency syndrome (AIDS) worldwide. HIV is an enveloped single-stranded RNA virus containing a template for reverse transcription into a double-stranded DNA which takes over the host genome replication machinery [13]. In HIV, the surface envelope glycoprotein (gp120) is a sphingolipid-binding domain that aids the docking of HIV-1 to the lipid rafts (plasma membrane microdomains). Once the host membrane sphingolipid binds to the sphingolipid-binding domain of HIV (gp120), it allows fusion between the host cell and the virus, a crucial step in HIV infection [76]. The presence of sphingomyelinase has been shown to prevent the entry of HIV into the cell [77]. Reduced viral infectivity by attenuation of viral entry into T cells and other immune cells such as monocytes resulting from the inhibition of sphingolipid biosynthesis in host cells has also been demonstrated [78,79]. High GM3 ganglioside in the plasma membrane of CD4^+^ T lymphocytes causes resistance to HIV-1 infection by preventing envelope-mediated fusion by altering the lateral association of HIV-1 receptors [80,81]. Other glycoproteins (galactosyl ceramide, GD3, Gb3) have also been reported to bind to HIV envelope gp120 [70]. Antibodies against galactocerebroside have been shown to inhibit HIV internalization [82]. Budding of HIV takes place in lipid rafts of the plasma membrane. The envelope of HIV contains sphingomyelin derivatives and glycosphingolipids (hexosylceramide, GM3, GM2, GM1) [78,83]. Ganglioside interaction with Siglec-1 (cell surface lectin attaches to sialyllactose moiety in glycosphingolipids on the viral envelope) causes capture of HIV in mature dendritic cells and macrophages, resulting in the formation of a reservoir called ‘virus-containing compartment’ for autologous T cells and for harboring the virus [84,85]. Therapeutics targeted against virus-containing compartments can reduce HIV transmission and may even eradicate HIV reservoirs [85].

### 6.11. Rhinoviruses

Rhinoviruses activate acid sphingomyelinase which generates sphingolipid ceramide to form ceramide-enriched membrane platforms [56,86]. Inhibiting acid sphingomyelinase in the cell prevents the creation of ceramide and the resulting platforms. This step also prevents the cell from being infected by the rhinovirus. Acid sphingomyelinase and ceramide raft pathways are important for rhinovirus infection, although the role of ceramide-enriched membrane platforms in viral invasion is unknown. Sphingomyelinase appeared to promote replication of rhinoviruses, and as with influenza virus, sphingomyelins are downregulated in the presence of rhinoviruses [56].

### 6.12. SARS-CoV-2

Several studies have focused on the role of sphingolipids in SARS-CoV-2 infection and multiple sphingolipids have been shown to aid in infection. The SARS-CoV-2 virus activates acid sphingomyelinase which triggers the release of ceramide to the cell membrane, a crucial step for infection [87]. Inhibiting this enzyme decreases SARS-CoV-2 infection. Another study found that glucosylceramide inhibitors were also able to inhibit the replication of SARS-CoV-2, indicating that the virus uses glucosylceramide for cellular infection [88]. Other sphingolipids can block SARS-CoV-2 from infecting the cell. SARS-CoV-2 uses the ACE2 receptor for cell entry and invasion, and the spike protein undergoes S-acylation and lipidation during disease pathogenesis [89,90]. Sphingosine can bind to these receptors as well, preventing the virus from entering the cell [91]. Levels of serum S1P are significantly decreased in patients with acute respiratory distress syndrome, and low serum S1P levels are associated with worse COVID-19 outcomes [92]. Acid sphingomyelinase was also shown to prevent SARS-CoV-2 replication [56]. These studies show that even within the SARS-CoV-2 virus, there is a huge amount of variation in how the virus interacts with sphingolipids. Any treatment for SARS-CoV-2 infection that focuses on regulating sphingolipids will have to be carefully selected and studied as different sphingolipids can have markedly different effects on the virus. One treatment that has potential is a series of specific antidepressants such as amitriptyline, imipramine, desipramine, fluoxetine, sertraline, escitalopram, and maprotiline [87,93]. These drugs inhibit the acid sphingomyelinase ceramide raft pathway and have shown promise in preventing infection, although more research is needed to confirm this. The wide range of interactions between various sphingolipids and the SARS-CoV-2 virus provides many opportunities for developing preventive therapies.

### 6.13. Rubella Virus

Rubella virus binds to sphingomyelin present in the host plasma membrane, rendering sphingolipids an important role in viral infection [94,95]. Sphingomyelin synthase 1 is essential for the entry of the virus into the host cell [95]. Sphingomyelin formed by sphingomyelin synthases contributes towards the formation of pores in the host cell membrane following viral binding, aiding in the entry process [95]. These studies highlight the important role sphingolipids play in the viral entry mechanism.

**Table 1:** Selected sphingolipids involved in viral infections.

Virus	Sphingolipid Targets/Component Involved	Effect
Ebola	Sphingomyelin	Promotion [72]
Ebola	Glucosylceramide	Promotion [55]
HIV	Sphingomyelin	Inhibition [77]
Influenza	Glucosylceramide	Promotion [55]
Influenza	Sphingomyelin	Inhibition [56]
JEV	Sphingomyelin	Promotion [62]
Measles	Glucosylceramide	Promotion [55]
Rhinovirus	Ceramide	Promotion [56,86]
Rhinovirus	Sphingomyelin	Promotion [56]
SARS-CoV-2	Ceramide	Promotion [55,87]
SARS-CoV-2	Glucosylceramide	Promotion [55,88]
SARS-CoV-2	Sphingomyelin	Inhibition [56]
SARS-CoV-2	Sphingosine	Inhibition [91]

**Table 2:** Action of viruses at various levels of sphingolipid metabolism.

Mechanism	Virus	Metabolites Involved
Alteration of lipid composition of cell surface for viral entry	Influenza virus	Sphingomyelin synthase 1 [46,47]
	Japanese encephalitis	Sphingomyelin synthase 1, Sphingomyelinase [61,62]
	Norovirus	Sphingomyelin, Ceramide synthesis [73,74]
	Zika virus	NS4B protein causing sphingolipid alteration [59]
	Dengue virus	Upregulation of sphingomyelin and ceramide [57]
	Ebola virus	Sphingomyelin [72]
	Hepatitis C virus	Sphingomyelin [68]
	Human immunodeficiency virus (HIV)	Gp120, Sphingomyelinase, GM ganglioside, Galactocerebroside [76,77,80,81,82]
	Rhinovirus	Sphingomyelinase, Ceramide [56]
	SARS-CoV-2	Glucosylceramide, Sphingomyelinase activation, Sphingosine binding to ACE receptor halting viral entry, Acid sphingomyelinase [87,88,91,93]
	Adenovirus	Ceramide, Sphingomyelin degradation altered [75]
	Rubella virus	Sphingomyelin synthase 1 [94,95]
Alteration of replication machinery	Influenza virus	Sphingomyelinase, Sphingosine kinase-1 [50,56]
	Zika virus	Glucosylceramide, Lactosylceramide [60]
	Japanese encephalitis virus	Sphingomyelinase activation [61,62]
	Hepatitis C virus	Sphingomyelin induced RNA polymerase [69,70,71]
	West Nile virus	Sphingomyelin [63]
	Rhinovirus	Sphingomyelinase [56]
	SARS-CoV-2	Acid sphingomyelinase [56]
Viral replication in endoplasmic reticulum	Dengue virus	Ganglioside GM3 [53]
Alteration in budding and release	Influenza virus	Neuraminidase trafficking aided by sphingomyelin synthesis [48]
	HIV	Sphingomyelin, Gangliosides [78,83]
	West Nile virus	Altered sphingomyelinase activity [64]

## 7. Targeting Sphingolipid Biomarkers for Vaccine or Therapeutic Development

Sphingolipids can serve as targets for vaccine or therapeutic development through understanding how they modulate immune responses. These lipids have also been explored as biomarkers for vaccine efficacy. In the context of vaccine development, the early administration of the Bacille Calmette–Guérin (BCG) vaccine in newborns has been implicated due to its association with metabolic shifts, particularly the robust production of sphingolipids such as S1P, sphingomyelin, N-acylsphingosine, and glucosylceramide [96,97]. These intricate molecules known for their diverse biological functions have also been observed to undergo alterations following vaccination against Francisella tularensis [98]. This link underscores the potential role of sphingolipid biomarkers in influencing and shaping immune responses to vaccination.

Furthermore, a plasma metabolomic analysis of immune responses to herpes zoster (shingles) Zostavax vaccine revealed a strong association with glycosphingolipid on day 3 post-vaccination [99]. Similarly, sphingolipid pathways, known to modulate the immune response, were also enriched in the serum of individuals exhibiting high-antibody response to an inactivated COVID-19 vaccine [100]. This emphasizes the relevance of sphingolipids as potential indicators of vaccine efficacy.

While plasma metabolomics and lipidomics provide an overview of the role of lipid metabolism in infections, they do not inform what metabolite correlates with a single cell subset. Technologies such as single-cell metabolomics and single-cell RNA sequencing could provide a deeper understanding of the role of sphingolipids in causing infections by dissecting immunometabolic mechanisms at the level of a single cell. These methods could also overcome the limitation of the metabolic flux technology employing Seahorse bioanalyzer to study cellular metabolism that captures only a limited number of metabolites. Most metabolites not captured through these assays could be potential targets or biomarkers in the sphingolipid pathway, providing novel insights into the role of lipid metabolism in infections.

## 8. Conclusions

Sphingolipids can have a multitude of different effects on viruses, depending on both the virus and the sphingolipid itself [101]. Influenza virus, SARS-CoV-2, and rhinovirus are all downregulated at different points in the cell entry process [56]. Sphingolipids have a strong association with disease progression in influenza infection [102]. SARS-CoV-2 results in increased levels of C16:0 ceramide and reduced levels of C18:1 ceramide and dihydrosphingosine [103]. Viruses such as HIV, rhinovirus, and measles virus are dependent on the sphingolipids present in the host cell membrane to gain cellular entry [104]. Ceramide may serve as a receptor for viral entry into host cells, and aid in forming sites for viral replication. Alpha-galactosylceramide may act as an immunomodulator during a viral infection [105]. Glucosylceramide aids in viral entry and contributes to higher infectivity with influenza [55]. Viral infections could result in high levels of sphingomyelin in the plasma [106,107]. Activation of SphK1/2 in response to a viral infection leads to clonal activation of CD8^+^ T cells [5]. However, viruses could exploit host SphK1/2 to replicate [108]. These mechanisms indicate that viruses can interact with sphingolipids at multiple points in the cell cycle. These sphingolipid metabolites may serve as biomarkers for predicting the severity of a viral infection. Clearly, no specific interaction is applicable to all sphingolipid-virus pairings.

Here, we have reviewed the known mechanisms of action of sphingolipids in viral infections and highlighted gaps that may be critical for the development of vaccines and antiviral agents. More work will be necessary to determine other ways in which sphingolipids and viruses interact, and the role this plays in infectious diseases.
